# Ambient fine particulate matter exposure induces reversible cardiac dysfunction and fibrosis in juvenile and older female mice

**DOI:** 10.1186/s12989-018-0264-2

**Published:** 2018-06-25

**Authors:** Guohua Qin, Jin Xia, Yingying Zhang, Lianghong Guo, Rui Chen, Nan Sang

**Affiliations:** 1 College of Environment and Resource, Research Center of Environment and Health, Shanxi University, Taiyuan, Shanxi 030006 People’s Republic of China; 2 State Key Laboratory of Environmental Chemistry and Ecotoxicology, Research Center for Eco-Environmental Sciences, Chinese Academy of Sciences, Beijing, 100085 People’s Republic of China; 3 CAS Key Laboratory for Biomedical Effects of Nanomaterials and Nanosafety& CAS Center for Excellence in Nanoscience, Beijing Key Laboratory of Ambient Particles Health Effects and PreventionTechniques, National Center for Nanoscience & Technology of China, Beijing, 100190 People’s Republic of China

**Keywords:** Particulate matter, Cardiac, Fibrosis, Reversible, Different age

## Abstract

**Background:**

Cardiovascular disease is the leading cause of mortality in the advanced world, and age is an important determinant of cardiac function. The purpose of the study is to determine whether the PM_2.5_-induced cardiac dysfunction is age-dependent and whether the adverse effects can be restored after PM_2.5_ exposure withdrawal.

**Methods:**

Female C57BL/6 mice at different ages (4-week-old, 4-month-old, and 10-month-old) received oropharyngeal aspiration of 3 mg/kg b.w. PM_2.5_ every other day for 4 weeks. Then, 10-month-old and 4-week-old mice were exposed to PM_2.5_ for 4 weeks and withdrawal PM_2.5_ 1 or 2 weeks. Heart rate and systolic blood pressure were measured using a tail-cuff system. Cardiac function was assessed by echocardiography. Left ventricles were processed for histology to assess myocardial fibrosis. ROS generation was detected by photocatalysis using 2′,7′-dichlorodihydrofluorescein diacetate (DCFHDA). The expression of cardiac fibrosis markers (Col1a1, Col3a1) and possible signaling molecules, including NADPH oxidase 4 (NOX-4), transforming growth factor β1 (TGFβ1), and Smad3, were detected by qPCR and/ or Western blot.

**Results:**

PM_2.5_ exposure induced cardiac diastolic dysfunction of mice, elevated the heart rate and blood pressure, developed cardiac systolic dysfunction of 10-month-old mice, and caused fibrosis in both 4-week-old and 10-month-old mice. PM_2.5_ exposure increased the expression of Col1a1, Col3a1, NOX-4, and TGFβ1, activated Smad3, and generated more reactive oxygen species in the myocardium of 4-week-old and 10-month-old mice. The withdrawal from PM_2.5_ exposure restored blood pressure, heart rate, cardiac function, expression of collagens, and malonaldehyde (MDA) levels in hearts of both 10-month-old and 4-week-old mice.

**Conclusion:**

Juvenile and older mice are more sensitive to PM_2.5_ than adults and suffer from cardiac dysfunction. PM_2.5_ exposure reversibly elevated heart rate and blood pressure, induced cardiac systolic dysfunction of older mice, and reversibly induced fibrosis in juvenile and older mice. The mechanism by which PM_2.5_ exposure resulted in cardiac lesions might involve oxidative stress, NADPH oxidase, TGFβ1, and Smad-dependent pathways.

**Electronic supplementary material:**

The online version of this article (10.1186/s12989-018-0264-2) contains supplementary material, which is available to authorized users.

## Background

Air pollution, mostly by fine particulate matter (PM_2.5_), leads to 3.3 million premature deaths per year worldwide, predominantly in Asia [1]. Epidemiological evidence supports a robust association between exposure to PM_2.5_ and cardiovascular diseases morbidity and mortality, such as myocardial infarction, heart failure, heart attacks, stroke, heart rhythm disturbances, and sudden death [2, 3]. However, the physicochemical properties of ambient PM_2.5_ in different regions varies because of a number of factors including local geography, proximity to emission sources, and meteorology. Even in the same region, PM_2.5_ from different seasons appears to have different chemical composition. It has been reported that seasonal variation in the association between PM_2.5_ and cardiovascular hospitalization [4]. Furthermore, PM_2.5_ does not affect all people equally. Several studies have suggested that susceptible individuals are at greater risk for PM_2.5_-associated cardiovascular morbidity and mortality including the elderly, women, and patients with preexisting coronary artery disease and diabetes [5, 6].

Recent studies have demonstrated that exposure to PM_2.5_ promotes systolic and diastolic dysfunction [7, 8], and exposure to carbon black impairs cardiac function in senescent mice [9]. Furthermore, exposure to diesel exhaust or PM_2.5_ during early life can cause significant cardiovascular dysfunction in adulthood [10, 11]. However, the susceptibility of individuals of different ages to cardiovascular disease caused by PM_2.5_ exposure has not been investigated. We hypothesized that PM_2.5_ exposure may induce different effects in different life phases, such as juvenile, adult, and older subpopulations.

The specific molecular mechanisms of PM_2.5_-induced cardiotoxicity effects are still under active investigation. Numerous investigations have elucidated potential biological mechanisms, whereby exposure to PM_2.5_ may modulate disease susceptibility, including the progression of atherosclerosis, inflammation, thrombosis, systemic vascular dysfunction, and epigenetic changes [12]. Cardiac fibrosis is a common phenotype found in several cardiac diseases, including myocardial infarction and heart failure. It is characterized by the adverse accumulation of collagen and other extracellular matrix proteins. In addition to the loss of contractile capacity, inhalation of PM_2.5_ is associated with adverse ventricular remodeling and worsening of cardiac fibrosis [7, 11]. Transforming growth factor β1 (TGFβ1), a critical regulator of fibroblast phenotype and function, acts through Smad-dependent or independent pathways [13]. However, the mechanisms underlying the cardiac fibrosis effects of PM_2.5_ are unclear.

Several previous studies of seasonal patterns of cardiovascular disease (CVD) indicated a peak of CVD in winter months [4, 14, 15]. We found seasonal variation in associations between collagen expression and PM_2.5_ exposure in H9C2 cells in our pilot experiment. The strongest effect on collagen expression was observed after treatment with PM_2.5_ from winter (Additional file 1: Figure S1). Accordingly, winter PM_2.5_ was used for in vivo experiments in mice in the present study. The dose of PM_2.5_ used in the present study was based on the following reasoning. As reported, respiratory volume of one mouse for 2 days reaches 0.259 m^3^ [16]. According to the Chinese ambient air quality secondary standards (GB3095–2012) of PM_2.5_ (75 μg/m^3^), the amount of PM_2.5_ inhalation for each mouse over 2 days is 19.425 μg. Therefore, PM_2.5_ exposure concentration for mouse (about 20 g b.w.) every 2 days should be 0.97 mg/kg (b.w.). The dose used in the present study was about 3 fold of secondary standards or more than 3.6 fold when considering the deposition fraction [17]. However, the PM_2.5_ concentration could exceed 300 μg/m^3^ during the polluted periods in Beijing [18] or other cities in China (http://113.108.142.147:20035/emcpublish/). The average concentration of PM_2.5_ in northern China with non-haze weather was 161 μg/m^3^ [19], and the level with haze weather reached 692 μg/m^3^ [20].

Therefore, the purpose of this study was: (1) to determine whether the PM_2.5_-induced cardiac dysfunction is age-dependent; (2) to examine whether the above-mentioned effects could be restored after PM_2.5_ exposure withdrawal; (3) to determine the potential mechanism of susceptibility to PM_2.5_ exposure.

## Results

### Exposure to PM_2.5_ elevates heart rate and systolic blood pressure of 10-month-old mice

**Fig. 1:**
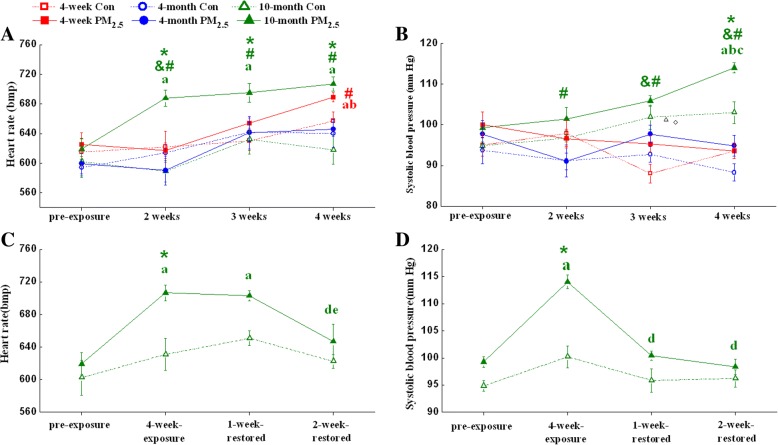
PM_2.5_ reversibly elevates heart rate and systolic blood pressure of mice. (**a**, **c**) Heart rate and (**b**, **d**) systolic blood pressure of mice exposed to PM_2.5_ (3 mg/kg b.w.).The data are expressed as the mean ± SE (*n* = 7). *, *P* < 0.05 vs. age-matched control; &, *P* < 0.05 vs. 4-week-old PM_2.5_ group; #, *P* < 0.05 vs. 4-month-old PM_2.5_ group; a, *P* < 0.05 vs. pre-exposure; b, *P* < 0.05 vs. 2-week-exposure; c, *P* < 0.05 vs. 3-week-exposure; d, *P* < 0.05 vs. 4-week-exposure; e, *P* < 0.05 vs. 1-week-restored group by two-way ANOVA and Turkey’s post hoc tests

For the heart rate of 4-week-old mice, 4 weeks of PM_2.5_ exposure caused a significant increase compared with pre-exposure but a non-significant increase compared with age-matched control group (Fig. 1a). The systolic blood pressure of 4-week-old mice was not affected by PM_2.5_ during 4 weeks of exposure (Fig. 1b). Neither heart rate nor the systolic blood pressure of 4-month-old mice was changed after PM_2.5_ exposure within the 4 weeks observation period. For 10-month-old mice, 2 weeks of PM_2.5_ exposure caused a significant increase in the heart rate compared with pre-exposure or age-matched control group (Fig. 1a). The heart rate of 10-month-old mice was significantly higher than 4-month-old mice after PM_2.5_ exposure (Fig. 1a). The systolic blood pressure of 10-month-old mice was elevated after 4 weeks of exposure compared with age-matched control group (Fig. 1b). The systolic blood pressure of 10-month-old mice was significantly higher than both 4-week-old and 4-month-old mice after PM_2.5_ exposure (Fig. 1b). Interestingly, the heart rate of 10-month-old mice decreased to base level after withdrawal from PM_2.5_ exposure for 2 weeks (Fig. 1c). The systolic blood pressure of 10-month-old mice was almost completely restored after withdrawal from PM_2.5_ exposure for only 1 week (Fig. 1d).

### Exposure to PM_2.5_ induces cardiac dysfunction in mice

**Fig. 2:**
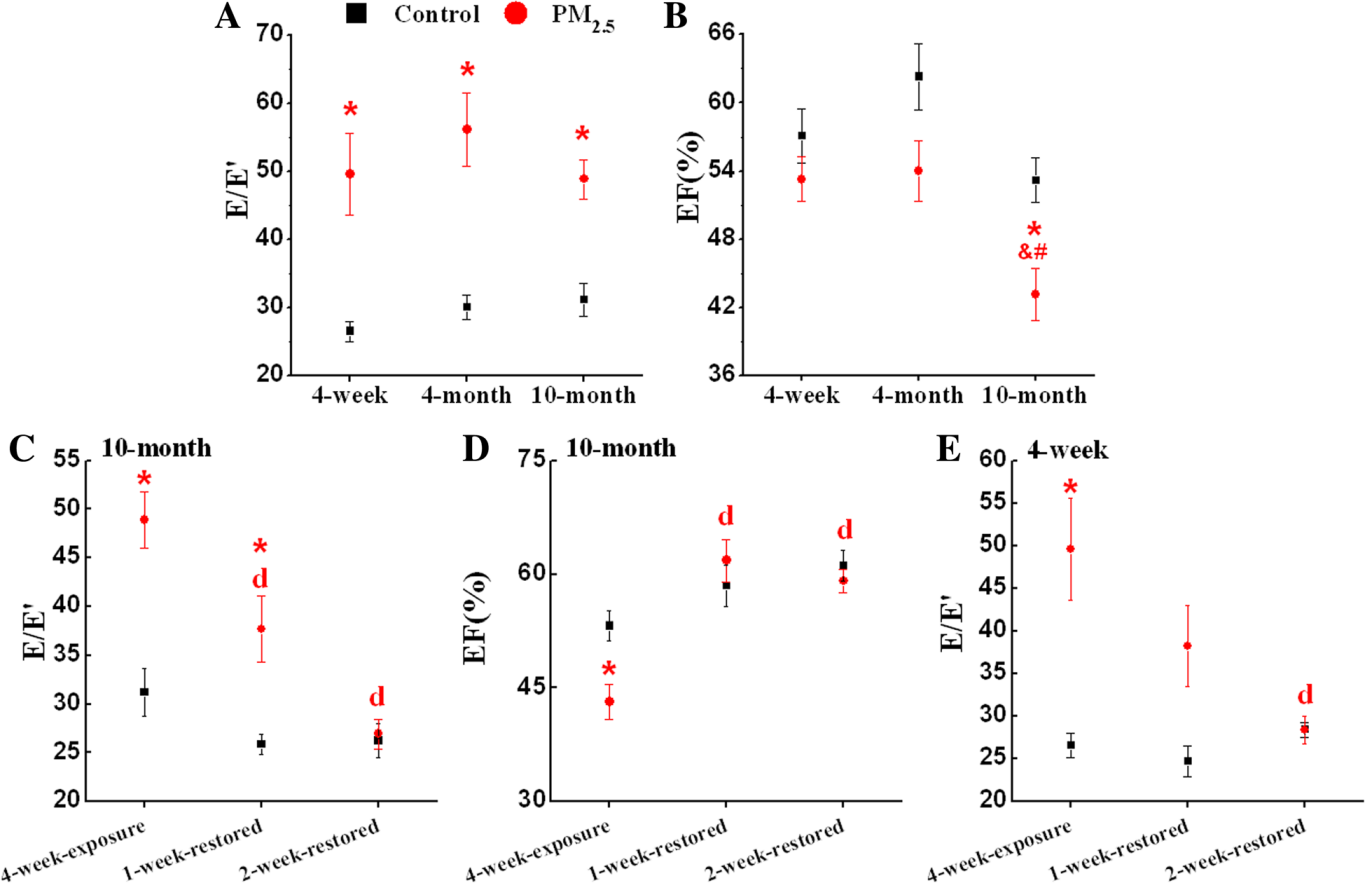
PM_2.5_ reversibly induces cardiac dysfunction in mice. (**a**) E/E’ of different age mice; (**b**) EF of different age mice; (**c**) E/E’ of 10-month-old mice; (**d**) EF of 10-month-old mice; (**e**) E/E’ of 4-week-old mice. The data are expressed as the mean ± SE (*n* = 6~ 9). E/E’, the ratio of peak early diastolic flow velocities and peak early motion wave values; EF, ejection fraction. *, *P* < 0.05 vs. age-matched control by two-way ANOVA and Turkey’s post hoc tests; &, *P* < 0.05 vs. 4-week-old PM_2.5_ group; #, *P* < 0.05 vs. 4-month-old PM_2.5_ group; d, *P* < 0.05 vs. 4-week-exposure by two-way ANOVA and Bonferroni’s post hoc tests

The data obtained from echocardiography are presented in Fig. 2. The ratio of peak early diastolic flow velocities and peak early motion wave values (E/E’) was significantly changed in mice at different ages after PM_2.5_ exposure (Fig. 2a). The E/E’ of 10-month-old mice and 4-week-old mice was restored after withdrawal from PM_2.5_ exposure for 2 weeks (Fig. 2c, e). PM_2.5_ exposure caused a significant decrease in the ejection fraction (EF) of 10-month-old mice compared to age-matched control group. (Fig. 2b). The EF of 10-month-old mice was significantly lower than both 4-week-old and 4-month-old mice after PM_2.5_ exposure (Fig. 2b). The EF of 10-month-old mice was restored after withdrawal from PM_2.5_ exposure for 1 week (Fig. 2d).

### Exposure to PM_2.5_ induces cardiac fibrosis in 4-week-old and 10-month-old mice

**Fig. 3:**
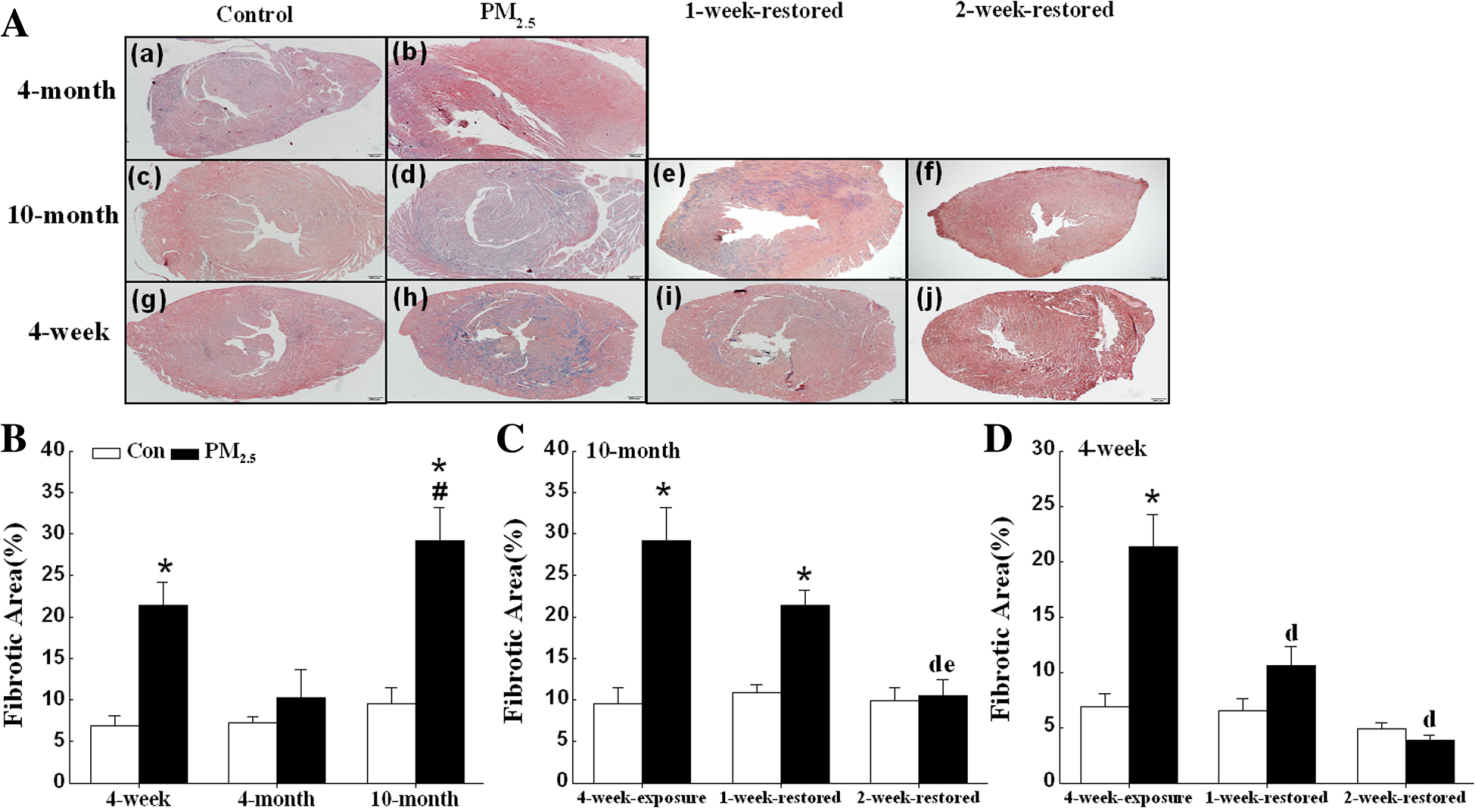
PM_2.5_ reversibly induces cardiac fibrosis of mice. Assessment of cardiac fibrosis by Masson’s Trichrome staining. (**a**) Representative images (4X objective) of hearts from mice exposed to PM_2.5_ (3 mg/kg b.w.) for 4 weeks, scale bar = 200 μm. The percentage of fibrotic regions of different ages mice (**b**), 10-month-old mice withdrawal of PM_2.5_ exposure (**c**), and 4-week-old mice withdrawal of PM_2.5_ exposure (**d**) was quantified. Fibrotic regions of cardiac tissues were determined by blue staining in each group. The data are expressed as the mean ± SE (*n* = 6~ 7). *, *P* < 0.05 vs. age-matched control by two-way ANOVA and Turkey’s post hoc tests; #, *P* < 0.05 vs. 4-month-old PM_2.5_ group; d, *P* < 0.05 vs. 4-week-exposure; e, *P* < 0.05 vs. 1-week-restored group by two-way ANOVA and Bonferroni’s post hoc tests

Histological examination of PM_2.5_-exposed mouse hearts stained with Masson’s trichrome revealed increased collagen deposition/myocardial fibrosis (Fig. 3). The hearts from PM_2.5_-exposed 4-week-old and 10-month-old mice showed interstitial fibrosis distributed diffusely across the left ventricular free wall (Fig. 3a). Quantification of the fibrotic area demonstrated that fibrosis was significantly greater in the PM_2.5_-exposed 4-week-old and 10-month-old mice than the corresponding control mice (Fig. 3b). The fibrotic area in hearts from 10-month-old mice was significantly greater than in hearts form 4-month-old mice after PM_2.5_ exposure (Fig. 3b). The fibrosis area reduced in 10-month-old mice after withdrawal from exposure to PM_2.5_ for 2 weeks (Fig. 3A, e-f; C). The fibrosis area was reduced in 4-week-old mice after withdrawal from exposure to PM_2.5_ for 2 weeks (Fig. 3A, i-j; D).

### Exposure to PM_2.5_ induces cardiac fibrosis marker expression in 4-week-old and 10-month-old mice

**Fig. 4:**
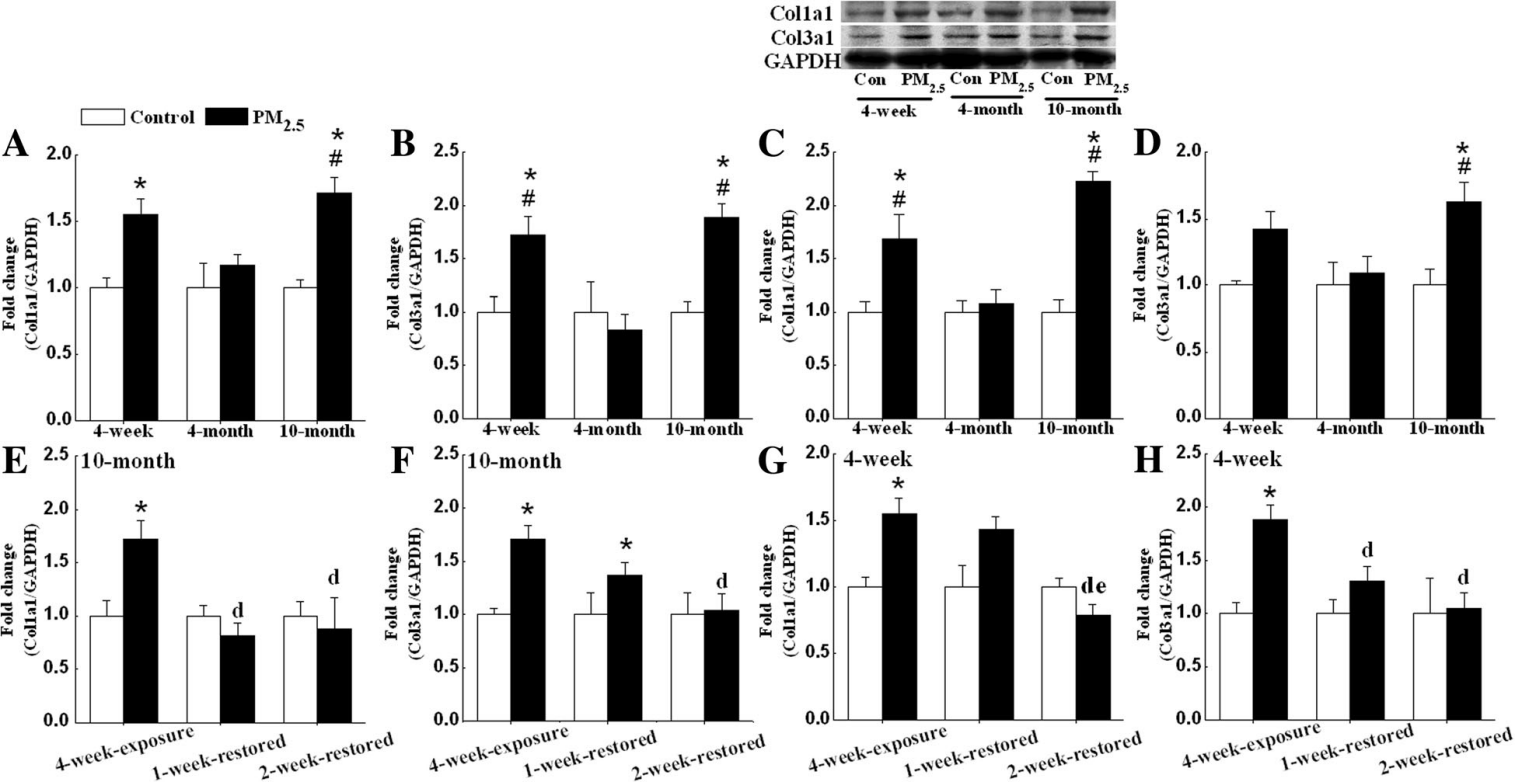
PM_2.5_ reversibly induces cardiac fibrosis marker expression of mice. mRNA expressions of Col1a1 and Col3a1 in different age mice (**a**, **b**), 10-month-old mice withdrawal of PM_2.5_ exposure (**e**, **f**) and 4-week-old mice withdrawal of PM_2.5_ exposure (**g**, **h**) were detected by qPCR. Protein expressions of Col1a1 (**c**) and Col3a1 (**d**) in different age mice were measured by Western blot. GAPDH was used as the internal control. The mean expression in each treated group was shown as a fold change compared to the mean expression in each control group, which had been calculated as target gene or protein /GAPDH and ascribed an arbitrary value of 1. Each column and bar represents the mean ± SE (n = 6~ 9). *, *P* < 0.05 vs. age-matched control by two-way ANOVA and Turkey’s post hoc tests; #, *P* < 0.05 vs. 4-month-old PM_2.5_ group; d, *P* < 0.05 vs. 4-week-exposure; e, *P* < 0.05 vs. 1-week-restored group by two-way ANOVA and Bonferroni’s post hoc tests

PM_2.5_ exposure of 4-week-old and 10-month-old mice led to increased transcription of Col1a1 and Col3a1, the major structural collagen in the myocardium, suggesting that PM_2.5_ exposure alters gene expression consistent with a profibrotic phenotype (Fig. 4a, b). Western blot analyses confirmed increased protein expression of Col1a1 and Col3a1 in PM_2.5_-exposed 10-month-old mice and Col1a1 in PM_2.5_-exposed 4-week-old (Fig. 4c, d). Furthermore, consistent with above results regarding cardiac dysfunction and collagen deposition, the expression of Col1a1 and Col3a1 of 10-month-old and 4-week-old mice were restored after withdrawal from exposure to PM_2.5_ for one or 2 weeks (Fig. 4c-f).

### PM_2.5_ induces ROS in hearts of 4-week-old and 10-month-old mice

**Fig. 5:**
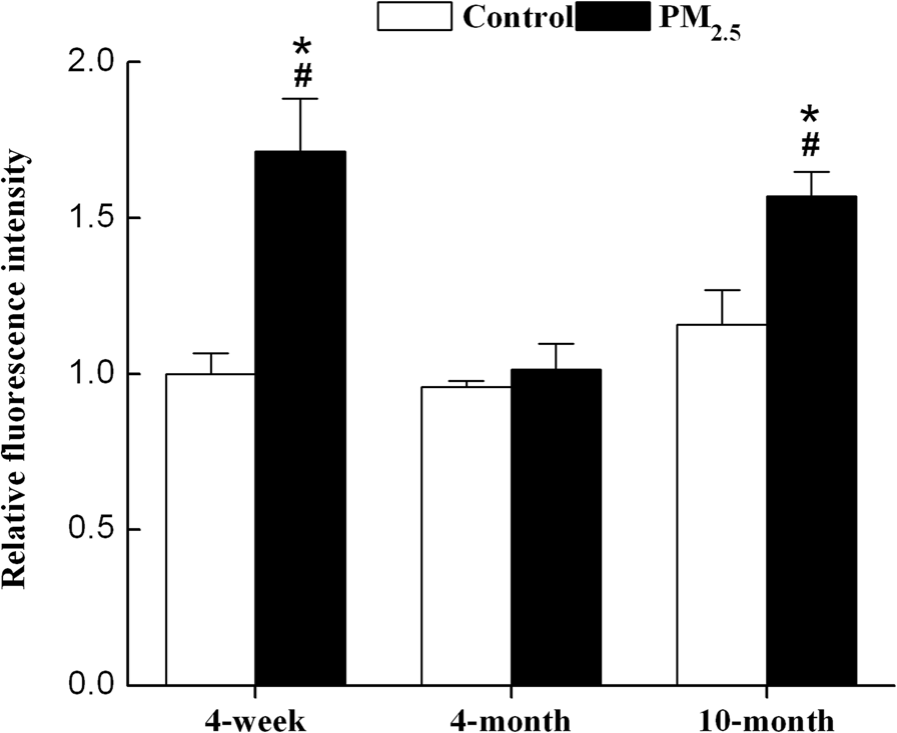
PM_2.5_ induces ROS generation in 4-week-old and 10-month-old mice. The generation of ROS by PM_2.5_ was detected by photocatalysis using the ROS-sensitive probe DCFHDA. Mean fluorescence units (FLU)/mg protein in each treated groups was shown as a fold change compared to the mean value in the control group of 4-week-old mice, which had been ascribed an arbitrary value of 1. Each column and bar represents the mean ± SE (n = 7~ 9). DCFHDA, 2′,7′-dichlorodihydrofluorescein diacetate. *, *P* < 0.05 vs. age-matched control by two-way ANOVA and Turkey’s post hoc tests. #, *P* < 0.05 vs. 4-month-old PM_2.5_ group by two-way ANOVA and Bonferroni’s post hoc tests

The generation of ROS induced by PM_2.5_ was detected by photocatalysis using an ROS-sensitive probe − 2′,7′- dichlorodihydrofluorescein diacetate (DCFHDA, Invitrogen). The ROS levels in the hearts of 4-week-old and 10-month-old mice exposed to PM_2.5_ were significantly increased compared to those of the corresponding control mice and 4-month-old mice after PM_2.5_ exposure (Fig. 5).

### PM_2.5_ induces inflammation and oxidative damage in hearts and lungs of 4-week-old and 10-month-old mice

**Fig. 6:**
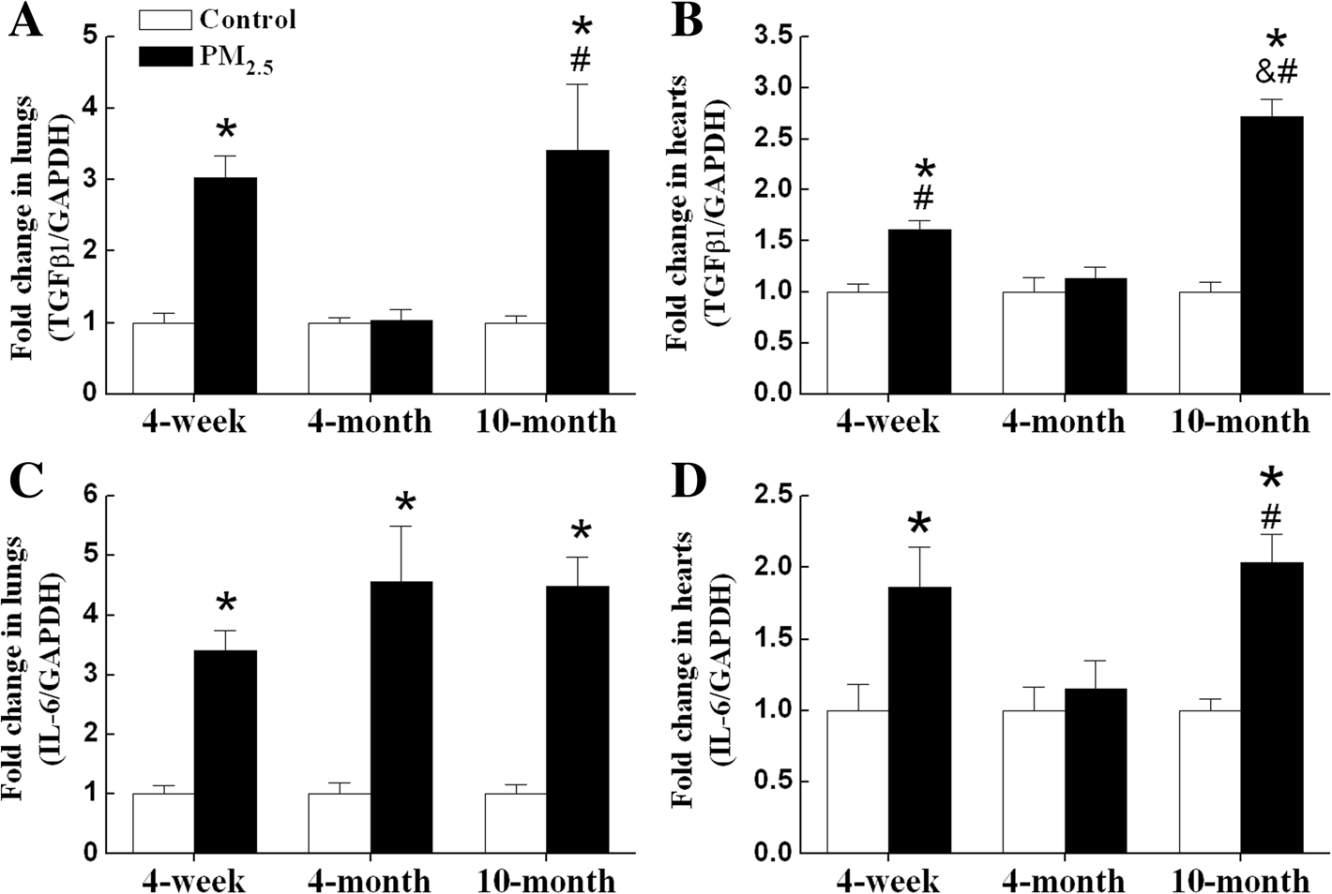
PM_2.5_ induces mRNA expression of TGFβ1 and IL-6 in lungs and hearts of mice. mRNA expression of TGFβ1 and IL-6 in lungs (**a**, **c**) and hearts (**b**, **d**) were detected by qPCR. GAPDH was used as the internal control. The mean expression in each treated group was shown as a fold change compared to the mean expression in each control group, which had been calculated as target gene /GAPDH and ascribed an arbitrary value of 1. Each column and bar represents the mean ± SE (n = 6–9). TGFβ1, transforming growth factor β1; IL-6, interleukin-6. *P < 0.05 vs. age-matched control by two-way ANOVA and Turkey’s post hoc tests; & *P* < 0.05 vs. 4-week-old PM_2.5_ group; # *P* < 0.05 vs. 4-month-old PM_2.5_ group by two-way ANOVA and Bonferroni’s post hoc tests

In order to indicate whether the occurrence of oxidative damage is a consequence of the local oxidative stress of lungs, we detected the mRNA levels of TGFβ1 and interleukin 6 (IL-6), and MDA levels in hearts and lungs. The mRNA levels of TGFβ1 were significantly increased in both lungs and hearts of 4-week-old and 10-month-old mice after PM_2.5_ exposure (Fig. 6a, b). The highest expression of TGFβ1 mRNA was observed in hearts of 10-month-old mice after PM_2.5_ exposure (Fig. 6b). The mRNA levels of IL-6 were significantly increased in lungs of all exposed groups mice and in hearts of 4-week-old and 10-month-old mice after PM_2.5_ exposure (Fig. 6c, d).

**Fig. 7:**
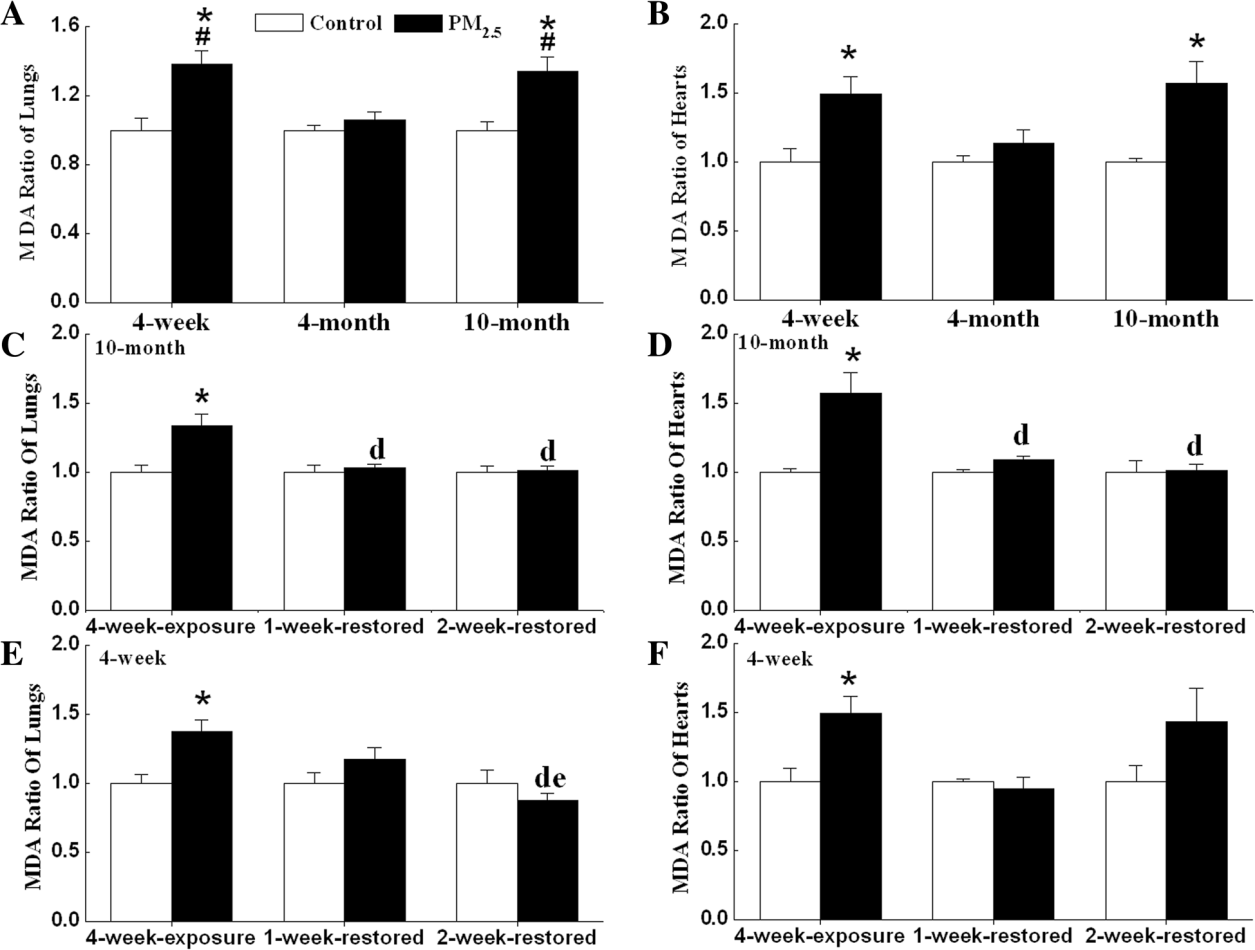
PM_2.5_ reversibly increases MDA levels in lungs and hearts of mice. MDA levels in different age mice (**a**, **b**), 10-month-old mice withdrawal of PM_2.5_ exposure (**c**, **d**) and 4-week-old mice withdrawal of PM_2.5_ exposure (**e**, **f**) were detected. Mean MDA levels in each treated groups was shown as a fold change compared to the mean value in each control group, which had been ascribed an arbitrary value of 1. Each column and bar represents the mean ± SE (n = 6~ 9). *, *P* < 0.05 vs. age-matched control by two-way ANOVA and Turkey’s post hoc tests; #, *P* < 0.05 vs. 4-month-exposure; d, *P* < 0.05 vs. 4-week-exposure; e, *P* < 0.05 vs. 1-week-restored group by two-way ANOVA and Bonferroni’s post hoc tests

The MDA levels in the hearts and lungs of 4-week-old and 10-month-old mice exposed to PM_2.5_ were significantly higher than in aged-matched controls (Fig. 7a, b). The MDA levels in hearts and lungs of 10-month-old mice decreased to base level after withdrawal from exposure to PM_2.5_ for 1 week (Fig. 7c-d). The MDA levels of 4-week-old mice did not change compared to corresponding controls after withdrawal from exposure to PM_2.5_ for one or 2 weeks (Fig. 7e-f).

### PM_2.5_-induced cardiac fibrosis is associated with NOX4-ROS-TGFβ1-Smad signaling

**Fig. 8:**
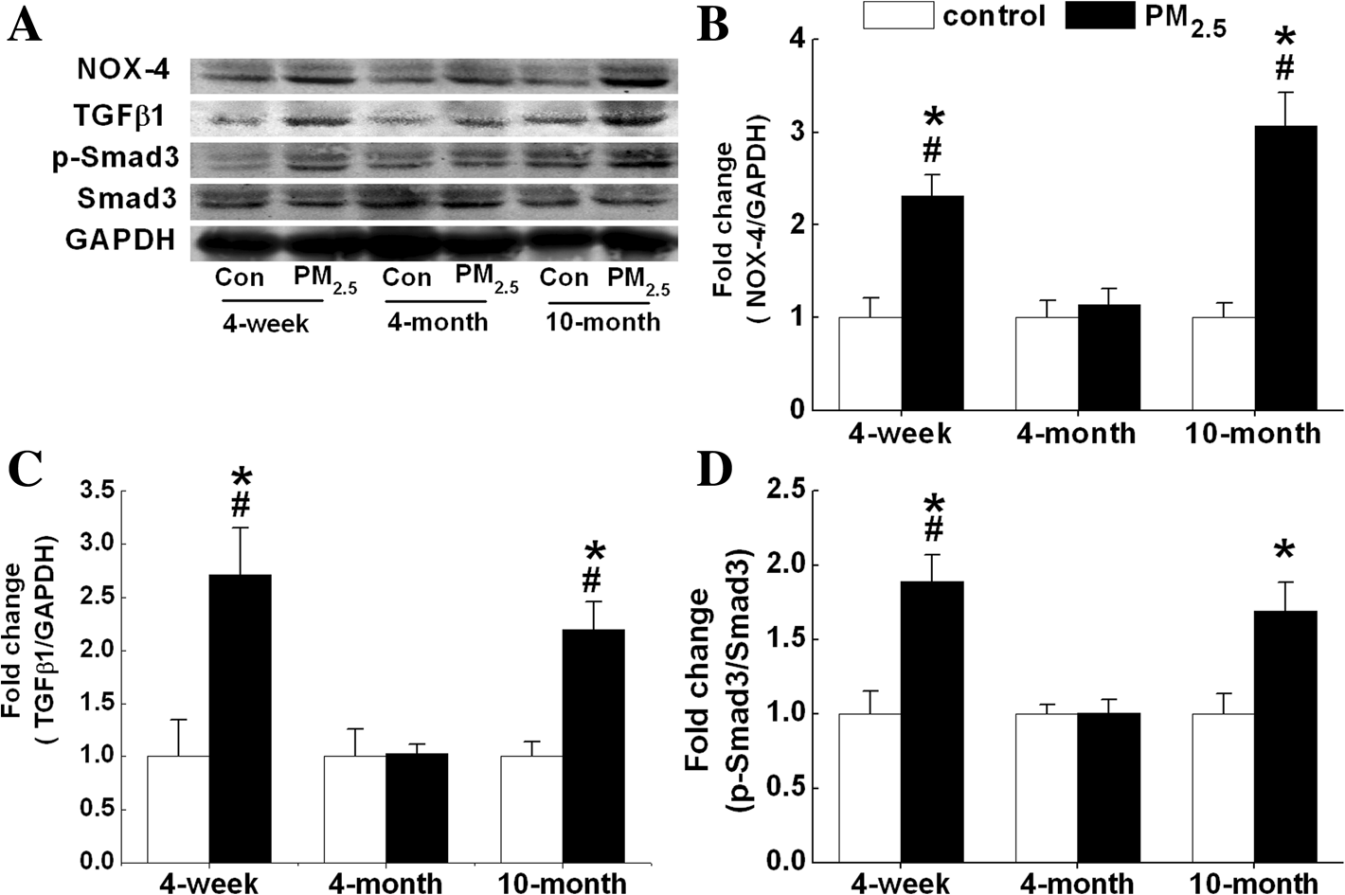
PM_2.5_-induced cardiac fibrosis is associated with NOX4-TGFβ1-Smad signaling. Protein levels were measured by Western blot. (**a**), Representative Western blot results of each protein. The protein levels of NOX-4 (**b**), TGFβ1 (**c**), and ratio of p-Smad3 and Smad 3 (**d**). Each column and bar represents the mean ± SE (*n* = 3~ 6). The mean expression in each treated group was shown as a fold change compared to the mean expression in each control group, which had been calculated as target protein /GAPDH and ascribed an arbitrary value of 1. NOX-4, NADPH oxidase 4; TGFβ1, transforming growth factor β1. *, *P* < 0.05 vs. age-matched control by two-way ANOVA and Turkey’s post hoc tests; #, *P* < 0.05 vs. 4-month-exposure by two-way ANOVA and Bonferroni’s post hoc tests

Western blotting was used to detect the protein expression of possible signaling molecules involved in PM_2.5_-induced cardiac fibrosis. NOX-4 and TGFβ1 protein levels were significantly increased in the hearts of 4-week-old and 10-month-old mice after PM_2.5_ exposure (Fig. 8a-c). After exposure for 4 weeks, PM_2.5_ treatment activated Smad3 in the hearts of 4-week-old and 10-month-old mice (Fig. 8a & d). No significant differences in the protein levels of NOX-4 or TGFβ1 or in the phosphorylation of Smad3 were observed in the hearts of 4-month-old mice compared with the control group (Fig. 8).

## Discussion

The results of our present study provide evidence that PM_2.5_ aspiration induced cardiac dysfunction and fibrosis in 4-week-old and 10-month-old mice, and the adverse effects could be resolved after PM_2.5_ exposure withdrawal. Cardiovascular disease is the leading cause of mortality in the advanced world, and age is an important determinant of cardiac function. The morbidity and mortality rates associated with PM_2.5_-induced cardiovascular disease are significantly higher in the elderly than in adults. Based on preliminary epidemiologic evidence, infants less than 2 years old and adults over 65 years old are the most susceptible subpopulations to PM [21]. Numerous findings support the association between PM exposure and electrocardiogram abnormalities in elderly subjects [22]. Several epidemiological studies have demonstrated associations between acute and chronic PM_2.5_ exposure and the elevation of heart rate and blood pressure, especially among elderly or susceptible individuals [5, 23, 24]. However, the effects of childhood air pollution exposure on cardiovascular risk in human populations are not well defined.

Most studies on the cardiac effects of PM are performed in susceptible models, such as hypertensive [25, 26], ligation of coronary arteries [27], and gene deficient animals [28, 29]. In the present study, 4-week-old, 4-month-old, and 10-month-old mice were employed as juvenile, adult, and older subpopulations, respectively. It is reported that during the juvenile stages, the valve, left atrial size, left ventricular end-diastolic dimension, aortic root, and ascending aorta dimensions show linear growth and, during the adult stages, the dimensions plateau [30]. There is evidence for atrial hypertrophy and dilatation, and LV wall thickness increases with age in older mice. Other age-dependent cellular changes reported in aging rodents include fibroblast proliferation, collagen accumulation, and interstitial fibrosis in both the atria and the ventricles [31]. Our data revealed that subchronic exposure to PM_2.5_ significantly increased the heart rate and systolic blood pressure in older mice. Furthermore, the PM_2.5_ effects on heart rate and blood pressure of 10-month-old mice were reversible. This finding is consistent with previous studies showing that chronic inhalation exposure to concentrated ambient particulate matter increased heart rate and blood pressure in C57BL/6 mice [7, 32]. Ying et al. reported that chronic exposure to concentrated ambient particulate matter (CAP) significantly increased blood pressure of spontaneously hypertensive rats, and withdrawal from CAP exposure restored blood pressure [26]. In combination, these data provide strong evidence that exposure to PM_2.5_ may have significant effects on elderly because they are more susceptible to cardiovascular disease.

Wold and colleagues reported that a 9-month inhalation exposure to CAP resulted in systolic and diastolic dysfunction [7]. In utero and early life exposure to diesel exhaust or PM_2.5_ induces adult cardiac dysfunction [10, 11]. In the present study, the hearts of PM_2.5_-exposed mice of different ages displayed a phenotype of diastolic dysfunction, indicated by the index of E/E’ ratios. The E/E’ ratio is a reliable index for detecting diastolic dysfunction [33]. EF is a basic parameter that assess systolic functions of the heart. Decreases in EF suggest that subchronic exposure to PM_2.5_ induced systolic dysfunctions only in 10-month-old mice. Furthermore, PM_2.5_-induced cardiac dysfunction of 10-month-old and 4-week-old mice could be restored after PM_2.5_ withdrawal. It is in accordance with previous study that withdrawal from CAP exposure restored cardiac function in spontaneously hypertensive rats [26]. Some pharmacological agents/genetic modification could reduce PM_2.5_-induced hypertension in rats [8, 34]. It indicated that PM_2.5_-induced cardiac dysfunction is reversible.

We found that 10-month-old and 4-week-old mice developed more fibrosis following PM_2.5_-exposure. The impairment in heart function observed in 4-week-old and 10-month-old mice correlated with increases of collagen deposition. The increases in fibrillar collagen in the heart interstitium contribute to tissue stiffness, and increases in collagen deposition may contribute to impaired heart function. We observed PM_2.5_-induced fibrosis changes in 4-week-old and 10-month-old mice accompanied by increases in the gene expression of major structural collagen types (Col1a1 and Col3a1) in the myocardium. Other studies have documented similar results with exposure to concentrated ambient PM [7, 35] and carbon black [9].

The heart rate, blood pressure, cardiac functional and structural changes, and molecular marker data showed that older mice are the most susceptible group to PM_2.5_. Similar results were observed with secondhand smoke or lipopolysaccharide (LPS) treatment. Secondhand smoke or LPS induce more cardiac dysfunction, fibrosis, inflammation, and oxidative stress in aged mice than in young mice [36, 37]. Aging-associated cardiac abnormalities are manifested as diastolic cardiac dysfunction, cardiac hypertrophy, and fibrosis, as well as impaired contractile function [38, 39]. The negative effects of PM_2.5_ on older mice might accelerate the development of aging. Furthermore, our data showed that 4-week-old mice were more susceptible than 4-month-old mice. PM_2.5_ exposure induced cardiac fibrosis, elevated Col1a1 and Col3a1 expression in 4-week-old mice, but not in 4-month-old mice. One possible reason for the difference in susceptibility to PM_2.5_ of mice is their differences in estrogen levels. It is well established that the incidence of cardiovascular disease is reduced in females prior to menopause, which may be due to estrogen levels [40]. Estrogen protects cardiomyocytes against angiotensin II-induced sensitization of hypertension [41]. In the present study, we observed that PM_2.5_ elevated non-significantly the estradiol 2 (E2) levels of 4-week-old and 4-month-old mice. The E2 levels of 10-month-old mice were lower than 4-month-old mice in PM_2.5_ groups (Additional file 2: Figure S2). It still needs further research to study whether male mice display different age-dependent responses from female mice. Another possible mechanism of PM_2.5_-induced cardiac functional changes might involve the role of ROS. Oxidative stress is increased in human heart failure and animal models of cardiac hypertrophy and fibrosis [42]. The heart consists of various types of cells, such as cardiomyocytes and nonmyocytes, including fibroblasts, endothelial cells, smooth muscle cells, and immune system–related cells [43]. ROS could be produced in cardiomyocytes or endothelial cells [44]. Our recent study has shown that PM_2.5_ dose-dependently induced ROS production in H9C2 cells [45]. In the present study, PM_2.5_ induced ROS generation in hearts and lungs of 4-week-old and 10-month-old mice. These observations indicated that ROS and oxidative stress might play important roles in PM_2.5_-induced cardiac fibrosis.

Different potential mechanisms for PM-associated cardiovascular disease have been hypothesized as either activated by direct effects of pollutants on the cardiovascular system, or by indirect effects due to pulmonary oxidative stress and inflammatory responses [46]. In the present study, we observed the elevation of TGFβ1 and IL-6 mRNA expression and MDA levels in lungs and hearts of 4-week-old and 10-month-old mice. Although this does not exclude the possibility of direct particle translocation, it is possible that PM_2.5_ triggers the release of soluble inflammatory mediators from the lungs and subsequently causes cardiac inflammation through systemic inflammatory responses. Similarly, it is reported that extra-pulmonary effects due to carbon nanoparticles inhalation are dominated by indirect effects (particle-cell interactions in the lung) rather than direct effects (translocated carbon nanoparticles) [47].

ROS generation has been detected in rat ventricular myocytes exposed to diesel exhaust, and it is likely mediated through NADPH oxidase-dependent pathways [48]. NADPH oxidases are multisubunit enzymes that are largely distributed within heart cells [49]. NOX-4 is essential for the differentiation of human cardiac fibroblasts into myofibroblasts [42]. A similar phenomenon was observed in the present study by the examination of NOX-4 expression and direct measurement of intracellular ROS as DCFHDA-fluorescence in mice. Following activation, membrane-bound NADPH oxidase initiates single-electron transfers to molecular oxygen, resulting in the formation of ROS [50]. Our data showed that NOX-4 expression was increased in 4-week-old and 10-month-old mice after PM_2.5_ exposure. The increase in NOX-4 expression was accompanied by the elevation of TGFβ1 protein expression. ROS stimulates the release and activation of cytokines, such as TGFβ1 [51]. TGF-β1 is expressed and released by many cell types, including cardiomyocytes, cardiac fibroblasts, and immune cells [52]. Cardiac tissue fibrosis in mice hearts is associated with an increased expression of the profibrotic marker TGF-β1 in cardiac macrophages [53]. TGFβ1 is upregulated in various experimental models of cardiac hypertrophy [54], and functional blockade of TGFβ1 prevents cardiac interstitial fibrosis induced by pressure overload in rats [55]. However, TGFβ1 upregulates the expression of NADPH oxidase and induces further ROS generation [42]. Hu and colleagues suggested positive feedback between TGFβ1, NADPH oxidase-mediated ROS generation, and LOX-1 [56]. Among numerous fibrotic signals, TGFβ1 is reported to be a key fibrogenic mediator. Smad2 and Smad3 are well-documented downstream mediators of TGFβ1-induced fibrosis, and activations of Smad2 and Smad3 are known to stimulate matrix-component synthesis, such as collagens [13]. TGF-β1 activates Smad-mediated signaling pathways on binding to TGF-β receptors on cardiomyocytes and cardiac fibroblasts [57]. In our study, Smad3 was activated in both 4-week-old and 10-month-old mice after PM_2.5_ exposure. This finding implied that PM_2.5_ induced cardiac fibrosis might involve the TGFβ1-Smad pathway.

Many studies have documented that Zn and Mn can enter systemic circulation and lead to lung fibrosis and cardiovascular disease [58–60]. In our latest study, we found that PM_2.5_-bound metals could reach and deposit in the heart with a developmental window-dependent property. The content of Zn increased significantly in hearts of 4-week-old and 10-month-old mice. The contents of Mn and Cd increased significantly in hearts of 10-month-old mice [61]. It is speculated that heavy metals, such as Zn, Mn, and Cd, might be responsible for PM_2.5_ induced cardiac toxicity. However, further experiments are needed to confirm the hypothesis.

There are several limitations to our study. The first limitation is the exposure method. Although oropharyngeal aspiration has been commonly used for experimental PM exposure [62–64]. Because it delivers a bolus high dose of particles to the lung, rather than repeated inhalation at lower doses, it may produce different effects in the lungs and hearts compare with exposure by inhalation. An additional limitation pertains to the low sensitivity of the tail-cuff method used to assay heart rate and blood pressure in this study. It is reported that tail-cuff readings are on average 39 mmHg lower than telemetric measurements for systolic blood pressure [65]. We exerted considerable care in the measurements with careful training of the mice. The data of each animal were measured as the mean of at least 5 successful measurements.

## Conclusion

In this report, we provide evidence that PM_2.5_ exposure induced cardiac dysfunction in mice at all ages tested, elevated heart rate and blood pressure in 10-month-old mice, and caused cardiac fibrosis in 10-month-old and 4-week-old mice. In combination, these data provide strong evidence that exposure to PM_2.5_ has significant effects on juveniles and the elderly because they are more susceptible to cardiovascular disease. Furthermore, all the above adverse effects in 10-month-old and 4-week-old mice could be restored after withdrawal PM_2.5_ exposure for 2 weeks. The mechanisms by which PM_2.5_ exposure resulted in cardiac lesions might involve the generation of oxidative stress and inflammation in lungs and the activation of TGFβ1-Smad pathway.

## Methods

### Collection of PM_2.5_ samples

Sampling was performed between 2012 and 2013 in Taiyuan, Northern China. The PM_2.5_ samples were collected onto quartz filters (Φ90 mm, Munktell, Falun, Dalarna, Sweden) with PM middle-volume air samplers (TH-150CIII, Wuhan, China). Details of sample collection and components analysis of PM_2.5_ have been previously described [66] and provided in Additional file 3: Table S1-Table S4 in Supporting information.

### Animals and exposure

Female C57BL/6 mice were purchased from the Junke Biological Engineering Co., LTD (Nanjing, China). Animals were housed in individual stainless steel cages under standard conditions (24 ± 2 °C and 50 ± 5% humidity) with a 12 h light–dark cycle in SPF conditions. The protocol of animal procedures was conducted in accordance with the National Institutes of Health Guide for the Care and Use of Laboratory Animals and was approved by the Institutional Animal Care and Use Committee of Shanxi University.

In *study-1*, C57BL/6 mice at 4 weeks, 4 months and 10 months were randomized into two subgroups. The mice received oropharyngeal aspiration of 0.9% saline (control groups) or 3 mg/kg. b.w. PM_2.5_ suspension (3 mg/ml, PM_2.5_ groups) 15–30 μl depend on the body weight of each animal every other day for 4 weeks. When not being treated, the mice had free access to standard food and water. Mice were sacrificed 24 h after the final exposure.

In *study-2*, C57BL/6 mice at 4-week-old or 10-month-old were received oropharyngeal aspiration of 0.9% saline (controls) or 3 mg/kg. b.w. PM_2.5_ suspension (PM_2.5_ groups) every other day for 4 weeks. Mice were sacrificed 24 h, 1 week, or 2 weeks after the final exposure, respectively. The hearts and lungs were removed, and all samples were fixed or quick frozen in liquid nitrogen and stored at − 80 °C.

### Heart rate and blood pressure assay

Heart rate and blood pressure were measured using a standard tail-cuff system (BP-2010A System, Softron). The blood pressure and heart rate data of each mice were measured as the mean of at least 5 successful measurements.

### Echocardiographic assessment

Heart function and structure were assessed by echocardiography using a Vevo 770™ ultrasound system (VisualSonics, Toronto, Ontario, Canada), which included a 21-MHz transducer. Mice were anesthetized with 2% isoflurane administered via a nose cone, were shaved from the chest, and were placed in the supine position on a hotplate. Echocardiography and off-line data analysis was performed by a single observer. The observer was blinded as to the age and group of mice being analyzed. Two-dimensional (2D) and M-mode echocardiography images were obtained from the parasternal region and viewed to measure left ventricular end-diastolic dimension (LVEDD), left ventricular end-systolic dimension (LVESD), posterior wall thickness in diastole (LVPWd), left ventricular end diastolic volume (LVEDV), and left ventricular end systolic volume (LVESV). Left ventricle filling parameters were assessed from the ends of mitral leaflets in apical quadrilocular image, and peak early (E) and after (A) diastolic flow velocities were measured. Tissue Doppler assessment was conducted on apical quadrilocular image. Peak early motion (E’) and peak after motion (A’) wave values were measured. Ejection fraction (EF), fractional shortening (FS), E/A ratio, E’/A’ ratio, and E/E’ ratio were obtained using defined calculations. Each parameter was measured in at least 3 cycles, and mean figures were utilized. All measurements were performed offline using the Vevo 770™ system software (VisualSonics, Toronto, Ontario, Canada).

### Histological analysis by Masson’s trichrome staining

The cardiac tissue from mice of each group was rapidly removed, washed several times with 0.1 μM phosphate buffered saline (PBS, pH 7.4), fixed in 10% formalin for 24 h at room temperature, dehydrated by graded ethanol, and embedded in paraffin. Sections (5–6 μm-thick) were deparaffinized with xylene, stained with Masson’s trichrome staining, and observed by microscopy. Quantitative planimetric analyses were performed on three successive sections per slide, and at least 7 sections from 3 consecutive slides per mouse were examined. Each image was digitized using a digital camera and analyzed under a research microscope (Olympus, Japan) using Image-Pro Plus software (version 5.0). The left ventricular cross-section collagen accumulation was quantified by the ratio of blue area to total myocardium area. All analyses were performed by an investigator blinded to the group assignments.

### ROS detection

Fresh hearts were used to prepare a 10% (*w*/*v*) PBS homogenate. After centrifuging at 1000 g for 10 min at 4 °C, the supernatant was collected and used to evaluate reactive oxygen species (ROS) and protein content. In 96-well plates, 190 μL of the supernatant and 10 μL of 1 mM DCFHDA were added to each well. After incubating at 37 °C for 30 min in the dark, the fluorescence was read at 485 nm for excitation and 530 nm for emission with a fluorescent plate reader (Varioskan Flash, Thermo Scientific, America). The results were calculated as the fluorescence units (FLU)/mg protein. The value of the 4-week-old mice in control group was set to 1.

### Oxidative stress analysis

The heart or lung tissues were homogenized and centrifuged, and the supernatants were collected for biomarker and protein concentration determination. The level of the lipid peroxidation product malondialdehyde (MDA) was measured using commercial kits (Nanjing Jiancheng Bioengineering Institute, Nanjing, China).

### Quantitative real-time PCR

Total RNA was isolated using TRIzol Reagent (TaKaRa, Dalian, China) according to the manufacturer’s instructions. Total RNA was then treated with DNase I (TaKaRa, Dalian, China), and cDNA was synthesized using a First Strand cDNA Synthesis kit (TaKaRa, Dalian, China) The cDNA product was stored at − 80 °C until used.

Real-time quantitative PCR (qPCR) was performed using an IQ5 Real-Time PCR System (Bio-Rad, USA) and SYBR Green qPCR Master Mix kit (Takara, Dalian, China). The primers are listed in Additional file 3: Table S5. The cycling conditions were as follows: 3 min at 95 °C, 40 cycles of 20 s at 94 °C, 20 s at 55–60 °C, and 20 s at 72 °C. The threshold cycle (Ct) values for the experimental samples were plotted onto the dilution series standard curve. The target quantities were calculated from separate standard curves generated for each experiment. The relative expression values were then determined by dividing the quantities of the target sequence of interest with the quantity obtained for GAPDH as an internal reference gene. The qPCR was repeated three times for each gene.

### Western blot analysis

Proteins were extracted in ice-cold lysis buffer (1% Nonidet P-40, 1 mM EDTA, 125 mM sodium fluoride, 0.5 mM sodium vanadate, 2.5 μg/mL of aprotinin, 5 μg/mL of pepstatin, 50 μg/mL of leupeptin, 25 μM PMSF, and 25 μg/mL of trypsin inhibitor). The protein concentration was determined according to the Bradford method using BSA as the standard protein [67]. Sodium dodecyl sulfate–polyacrylamide gel electrophoresis was performed on 50 μg protein samples using 12% resolving/4% stacking Tris-HCl gels. Electrophoresis proteins were transferred to nitrocellulose membranes using Bio-Rad Mini Trans-Blot Electrophoretic Transfer Cell Instruments. Membranes were blocked in 3% BSA solution for 1 h at room temperature and incubated overnight at 4 °C with antibodies to targeted proteins (anti-GAPDH antibody, CST; anti-Col1a1 antibody, Bioss; anti-Col3a1 antibody, Bioss; anti-TGFβ1 antibody, Proteintech; anti- NADPH oxidase 4 (NOX-4)antibody, BBI; anti-Smad 3 antibody, BBI; and anti-P-Smad 3 antibody, BBI). After washing, the membranes were incubated with fluorescently labeled secondary antibody (1,5000) (IRDye 800CW goat anti-rabbit IgG (H + L), LI-COR), scanned, and detected with the LI-COR Odyssey Infrared Fluorescent System.

### Statistical analysis

All data are expressed as the means ± standard error of the mean (SE). Comparison between groups in Fig. 1a and b was conducted using two-way repeated analysis of variance (ANOVA) followed by Tukey’s post-test within each x-axis time-point across all groups or Tukey’s post-test within each treatment group across x-axis time-points. Two-way non-repeated ANOVA followed by Tukey’s post-test within each group across the x-axis time-points or Bonferroni’s post-test within each x-axis time-point across the control and PM_2.5_ groups were used to compare between groups in others figures. Differences for all tests were considered significant when *P* < 0.05.

## Additional files

Additional file 1: **Figure S1.** PM_2.5_ induces collagen mRNA (A) and protein (B) expression in H9C2 cells. (A) mRNA expressions of Col1a1 and Col3a1 in H9C2 cells were detected by qPCR. (B) The protein levels of Col1a1 and Col3a1 were detected by Western blot. GAPDH was used as the internal control. The mean expression in four seasons PM_2.5_ treated group was shown as a fold change compared to the mean expression of control group, which had been calculated as target gene or protein /GAPDH and ascribed an arbitrary value of 1. Each column and bar represents the mean ± SE (n=3). Significantly different from control by one-way ANOVA with Tukey's post-test. * P<0.05 vs. control group; a P<0.05 vs. spring PM_2.5_ group; b P<0.05 vs. summer PM_2.5_ group; c P<0.05 vs. autumn PM_2.5_ group. (TIF 440 kb)

Additional file 2: **Figure S2.** Estrogen levels in plasma of different age mice. Each column and bar represents the mean ± SE (n=6). # *P*<0.05 vs. 4-week-exposure by two-way ANOVA and Bonferroni's post hoc tests. (TIF 106 kb)

Additional file 3: **Table S1.** Contents of inorganic ion in PM_2.5_ samples from different seasons (unit: μg/m^3^). **Table S2.** Element contents in PM_2.5_ samples from different seasons (unit: ng/m^3^). **Table S3.** Contents of polycyclic aromatic hydrocarbons (PAHs) in PM_2.5_ samples from different seasons (unit: ng/m^3^). **Table S4.** Carbon contents in PM_2.5_ samples from different seasons (unit: μg/m^3^). **Table S5.** Sequences of primers used in real-time PCR. (DOCX 35 kb)

## Abbreviations

ANOVA: Analysis of variance
bpm: Beats per minute
cDNA: Complimentary deoxyribonucleic acid
Col1a1: Collagen I
Col3a1: Collagen III
DCFHDA: 2′,7′- dichlorodihydro fluorescein diacetate
DMEM: Dulbecco’s modified eagle’s medium
E/E’: Ratio of peak early diastolic flow velocities and peak early motion wave values
EF: Ejection fraction
GAPDH: Glyceraldehye 3-phosphatede hydrogenase
IL-6: Interleukin 6
LPS: Lipopolysaccharide
LV: Left ventricle
mRNA: Messenger ribonucleic acid
NOX-4: NADPH oxidase subunit 4
PM: Particulate matter
PM_2.5_: Particulate matter with a diameter of 2.5 μm or less
qRT-PCR: Quantitative real time polymerase chain reaction
ROS: Reactive oxygen species
TGFβ1: Transforming growth factor β1
