# Supplementary figures and images for: Ambient fine particulate matter exposure induces reversible cardiac dysfunction and fibrosis in juvenile and older female mice
Source: Part Fibre Toxicol. 2018 Jun 25;15:27. doi: 10.1186/s12989-018-0264-2 (PMC6019275; doi:10.1186/s12989-018-0264-2)

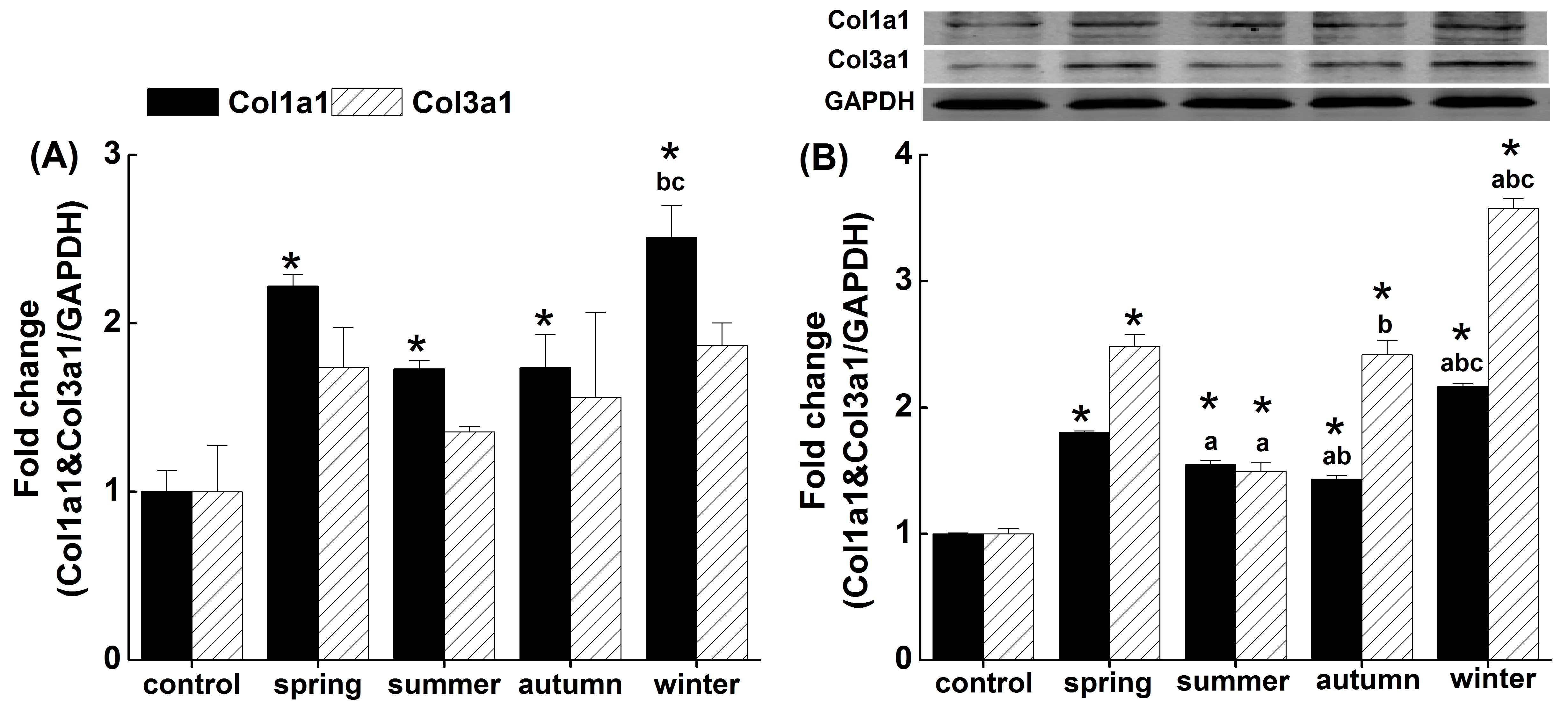

Supplement: Supplementary file 1 — Figure S1. PM2.5 induces collagen mRNA (A) and protein (B) expression in H9C2 cells. (A) mRNA expressions of Col1a1 and Col3a1 in H9C2 cells were detected by qPCR. (B) The protein levels of Col1a1 and Col3a1 were detected by Western blot. GAPDH was used as the internal control. The mean expression in four seasons PM2.5 treated group was shown as a fold change compared to the mean expression of control group, which had been calculated as target gene or protein /GAPDH and ascribed an arbitrary value of 1. Each column and bar represents the mean ± SE (n=3). Significantly different from control by one-way ANOVA with Tukey's post-test. * P<0.05 vs. control group; a P<0.05 vs. spring PM2.5 group; b P<0.05 vs. summer PM2.5 group; c P<0.05 vs. autumn PM2.5 group. (TIF 440 kb) [file 12989_2018_264_MOESM1_ESM.tif]

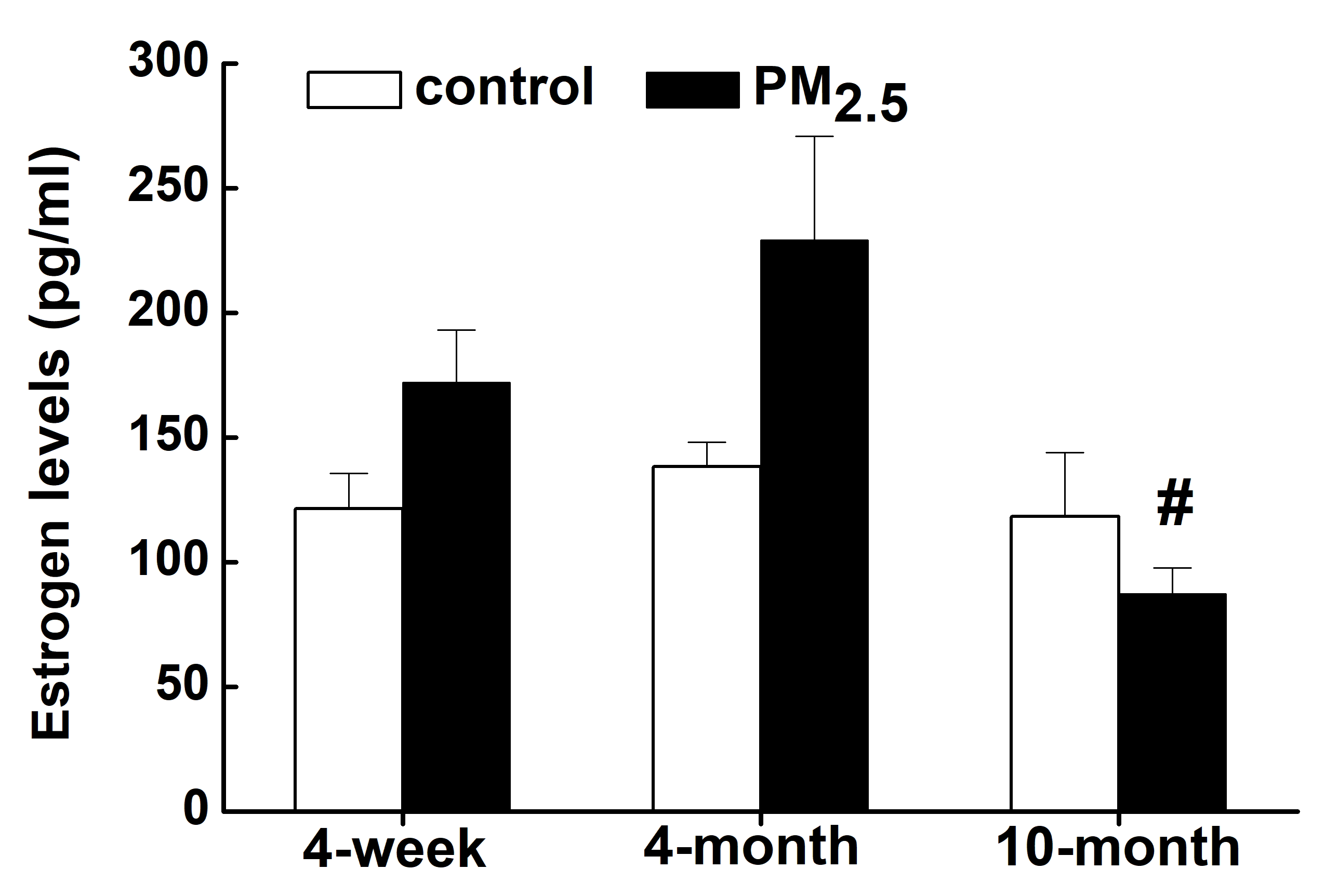

Supplement: Supplementary file 2 — Figure S2. Estrogen levels in plasma of different age mice. Each column and bar represents the mean ± SE (n=6). # P<0.05 vs. 4-week-exposure by two-way ANOVA and Bonferroni's post hoc tests. (TIF 106 kb) [file 12989_2018_264_MOESM2_ESM.tif]
