# Supplementary material for: Ambient fine particulate matter exposure induces reversible cardiac dysfunction and fibrosis in juvenile and older female mice
Source: Part Fibre Toxicol. 2018 Jun 25;15:27. doi: 10.1186/s12989-018-0264-2 (PMC6019275; doi:10.1186/s12989-018-0264-2)
Supplement: Supplementary file 3 — Table S1. Contents of inorganic ion in PM2.5 samples from different seasons (unit: μg/m3). Table S2. Element contents in PM2.5 samples from different seasons (unit: ng/m3). Table S3. Contents of polycyclic aromatic hydrocarbons (PAHs) in PM2.5 samples from different seasons (unit: ng/m3). Table S4. Carbon contents in PM2.5 samples from different seasons (unit: μg/m3). Table S5. Sequences of primers used in real-time PCR. (DOCX 35 kb) [file 12989_2018_264_MOESM3_ESM.docx]

**Cover Sheet**

| Manuscript Title | Ambient fine particulate matter exposure induces reversible cardiac dysfunction and fibrosis in juvenile and older female mice |
| --- | --- |
| Complete List of Authors | Guohua Qin^a^, Jin Xia^a^, Yingying Zhang^a^, Lianghong Guo^b^, Rui Chen^c^, Nan Sang^a,^* |
| Total Pages | 7 |
| Total Tables | 5 |
| Total Figures | 2 |

**Table S1** Contents of inorganic ion in PM_2.5_ samples from different seasons (unit: μg/m^3^).

| Inorganic ion | Spring | Summer | Autumn | Winter |
| --- | --- | --- | --- | --- |
| F ^-^ | 0.026 | 0.021 | 0.013 | 0.030 |
| Cl ^-^ | 0.092 | 0.024 | 0.035 | 0.345 |
| NO_3_ ^-^ | 0.174 | 0.189 | 0.153 | 0.088 |
| SO_4_^2 -^ | 0.235 | 0.463 | 0.388 | 0.282 |
| Na ^+^ | 4.093 | 3.400 | 3.403 | 3.409 |
| NH_4_ ^+^ | 4.901 | 6.369 | 6.216 | 6.755 |
| K ^+^ | 1.739 | 1.735 | 1.737 | 2.031 |
| Mg^2 +^ | 0.185 | 0.242 | 0.193 | 0.240 |
| Ca^2 +^ | 2.904 | 5.863 | 2.679 | 2.336 |

**Table S2** Element contents in PM_2.5_ samples from different seasons (unit: ng/m^3^).

| Element | Spring | Summer | Autumn | Winter |
| --- | --- | --- | --- | --- |
| Zr | 341.414 | 496.970 | 367.677 | 379.798 |
| Al | 30909.091 | 33393.939 | 32464.646 | 31313.131 |
| Sr | 202.020 | 182.424 | 122.424 | 141.212 |
| Mg | 11434.343 | 14767.677 | 14787.879 | 13737.374 |
| Ti | 1290.909 | 1444.444 | 1092.929 | 1143.434 |
| Ca | 14323.232 | 14848.485 | 10969.697 | 12585.859 |
| Fe | 2404.040 | 3151.515 | 2323.232 | 2262.626 |
| Ba | 5131.313 | 2343.434 | 1385.859 | 1301.010 |
| Li | 62.626 | 93.333 | 100.404 | 80.000 |
| Be | 0.768 | 2.424 | 2.626 | 2.020 |
| Na | 37171.717 | 11797.980 | 13373.737 | 14989.899 |
| P | 258.586 | 397.980 | 387.879 | 321.212 |
| K | 15353.535 | 7797.980 | 8161.616 | 8222.222 |
| Sc | 5.253 | 7.677 | 8.081 | 6.061 |
| V | 33.131 | 60.202 | 61.010 | 45.253 |
| Cr | 57.374 | 57.172 | 53.535 | 41.616 |
| Mn | 165.051 | 168.485 | 161.616 | 111.554 |
| Co | 3.232 | 2.626 | 1.717 | 1.374 |
| Ni | 16.566 | 14.141 | 6.667 | 5.051 |
| Cu | 21.414 | 23.636 | 19.596 | 16.845 |
| Zn | 310.783 | 235.354 | 303.030 | 393.939 |
| Rb | 17.778 | 20.000 | 16.566 | 13.131 |
| Y | 9.495 | 12.525 | 12.727 | 10.101 |
| Mo | 2.828 | 2.828 | 3.030 | 2.828 |
| Cd | 1.697 | 1.717 | 2.020 | 2.020 |
| Sn | 11.111 | 4.444 | 4.848 | 7.273 |
| Sb | 10.707 | 25.051 | 28.889 | 25.253 |
| Cs | 3.434 | 4.646 | 4.646 | 3.232 |
| La | 16.768 | 26.061 | 28.081 | 21.818 |
| Ce | 85.657 | 174.747 | 230.303 | 292.929 |
| Sm | 1.616 | 2.424 | 2.424 | 1.919 |
| W | 2.828 | 4.444 | 5.859 | 5.051 |
| Tl | 1.091 | 0.869 | 0.828 | 1.212 |
| Pb | 120.404 | 112.525 | 115.556 | 127.071 |
| Bi | 2.424 | 2.222 | 4.242 | 2.828 |
| Th | 6.263 | 9.495 | 10.101 | 8.687 |
| U | 2.222 | 3.232 | 3.434 | 2.828 |

**Table S3** Contents of polycyclic aromatic hydrocarbons (PAHs) in PM_2.5_ samples from different seasons (unit: ng/m^3^).

| PAHs | Spring | Summer | Autumn | Winter |
| --- | --- | --- | --- | --- |
| Naphthalene (NA) | 0.124 | 0.116 | 0.103 | 0.222 |
| Acenaphthylene (ACL) | 0.072 | 0.131 | 0.103 | 0.175 |
| Acenaphthene (AC) | 0.525 | 0.332 | 0.064 | 0.451 |
| Fluorene (FLU) | 0.459 | 0.239 | 0.348 | 1.672 |
| Benzo[g,h,i]pyrene (BPE) | 14.486 | 6.740 | 7.879 | 33.465 |
| Indeno[1,2,3-cd]pyrene (IPY) | 12.586 | 6.362 | 7.746 | 31.963 |
| Dibenzo[a,h]anthracene (DBA) | 4.509 | 2.203 | 2.654 | 9.986 |
| Benzo[b]fluoranthene (BbF) | 20.740 | 10.010 | 12.849 | 54.607 |
| Coronene (COR) | 6.686 | 3.632 | 4.314 | 10.189 |
| Phenanthrene (PHE) | 1.941 | 1.540 | 2.369 | 8.881 |
| Anthracene (ANT) | 0.280 | 0.214 | 0.287 | 1.708 |
| Fluoranthene (FA) | 5.941 | 3.803 | 5.396 | 38.023 |
| Benzo[a]anthracene (BaA) | 8.020 | 2.645 | 4.004 | 38.438 |
| Chrysene (CHR) | 12.109 | 5.182 | 7.854 | 39.493 |
| Pyrene (PYR) | 3.810 | 2.360 | 3.349 | 26.354 |
| Benzo[a]pyrene (BaP) | 8.031 | 3.037 | 3.863 | 32.636 |
| Benzo[e]pyrene (BeP) | 11.182 | 4.886 | 6.022 | 31.248 |
| Benzo[k]fluoranthene (BkF) | 4.710 | 2.171 | 2.445 | 16.456 |

**Table S4** Carbon contents in PM_2.5_ samples from different seasons (unit: μg/m^3^).

| Carbon | spring | summer | autumn | winter |
| --- | --- | --- | --- | --- |
| OC | 21.759 | 8.847 | 24.368 | 45.957 |
| EC | 9.651 | 5.220 | 11.601 | 15.476 |
| TC | 31.410 | 14.068 | 35.969 | 61.432 |

**Table S5 Sequences of primers used in real-time PCR**

| Gene | Species | Accession No. |  | sequences |
| --- | --- | --- | --- | --- |
| GAPDH  Col1a1  Col3a1  TGFβ1  IL-6 | Mice  Rats  Mice  Rats  Mice  Rats  Mice  Mice | NM_008084  NM_017008  NM_007742  NM_053304.1  NM_009930.2  NM_032085  NM_011577.1  NM_031168.2 | Sense  Antisense  Sense  Antisense  Sense  Antisense  Sense  Antisense  Sense  Antisense  Sense  Antisense  Sense  Antisense  Sense  Antisense | 5'-CTTTGGCATTGTGGAAGGGC-3'  5'-CAGGGATGATGTTCTGGGCA-3'  5'-ATGTATCCGTTGTGGATCTGAC-3'  5'-CCTGCTTCACCACCTTCTTG-3'  5'- CAGTCGCTTCACCTACAGCA-3'  5'- GGGTGGAGGGAGTTTACACG-3'  5′- GTACATCAGCCCAAACCCCA-3′  5′- ACAAGCGTGCTGTAGGTGAA-3′  5′- TCACCAGGACAAAGAGGGGA-3′  5′- CCACCAGGACTGCCGTTATT-3′  5'- GCTCGGAATTGCAGAGACCT-3'  5'- AGCATCCATCTTGCAGCCTT -3'  5'-CGTCAGACATTCGGGAAGCA -3'  5'-TGCCGTACAACTCCAGTGAC -3'  5'-GGGACTGATGCTGGTGACAA -3'  5'-ACAGGTCTGTTGGGAGTGGT -3' |

**Materials and methods**

**Cell culture and exposure**

Rat H9C2 transformed cardiomyoblasts were maintained in Dulbecco's modified Eagle's medium (DMEM) supplemented with 10% fetal bovine serum (FBS, Invitrogen) at 37°C and 5% CO_2_. Cells were treated with PM_2.5_ (10 μg/mL) collected from different seasons for 24 h.

**Estradiol 2 assay**

Estradiol 2 (E2) hormone level was determined using an Enzyme Linked Immunosorbent Assay Kit (Elabscience, Wuhan, China) and measured the absorbance at 450 nm.

**Statistical analysis**

All data are expressed as the means ± standard error of the mean (SE). Comparison between groups was conducted using one-way analysis of variance (ANOVA) followed by Tukey's post-test in Figure S1. Two-way ANOVA followed by Tukey's post-test within each group across the x-axis time-points or Bonferroni's post-test within each x-axis time-point across the control and PM_2.5_ groups were used to compare between groups in Figure S2.

**Results**

**Season-dependent effects of PM_2.5_ on collagen expression *in vitro***

We assayed the mRNA and protein levels of cardiac fibrosis markers in H9C2 cells. As shown in Fig. S1, the mRNA and protein levels of collagen I (Col1a1), as well as the protein levels of collagen III (Col3a1), were significantly elevated after exposure to 10 μg/mL PM_2.5_ from four seasons for 24 h (Fig. S1). Winter PM_2.5_ induced the strongest elevation of Col1a1 mRNA and protein and Col3a1 protein expression in H9C2 cells. According to above results *in vitro*, winter PM_2.5_ was chosen to expose to mice at different ages. As shown in Table S1-S4, more PAHs, carbon, and Zn in winter PM_2.5_ were detected. PAHs such as phenanthrene exposure increases cardiac fiber content [[1](#_ENREF_1)]. It has been documented that Zn can enter systemic circulation and lead to lung fibrosis and cardiovascular disease [[2](#_ENREF_2)]. It indicated that these components might be related to collagen deposition.

**Estrogen levels in plasma of different age mice**

PM_2.5_ elevated non-significantly the E2 levels of 4-week-old and 4-month-old mice. No significant differences were observed in control mice at different ages. However, E2 levels of 10-month-old mice were lower than 4-month-old mice in PM_2.5_ groups (Figure S2).

**References**

1 Zhang Y, Huang L, Wang C, Gao D, Zuo Z: **Phenanthrene exposure produces cardiac defects during embryo development of zebrafish (danio rerio) through activation of mmp-9**. *Chemosphere* 2013;**93**:1168-1175.

2 Kodavanti UP, Schladweiler MC, Gilmour PS, Wallenborn JG, Mandavilli BS, Ledbetter AD, Christiani DC, Runge MS, Karoly ED, Costa DL: **The role of particulate matter-associated zinc in cardiac injury in rats**. *Environmental Health Perspectives* 2008;**116**:13-20.
